# A Methodological Review of Drug-Related Toxicological Studies in Saudi Arabia

**DOI:** 10.7759/cureus.36369

**Published:** 2023-03-19

**Authors:** Hassan Alwafi, Rayan Khayat, Alaa Banjabi, Emad Salawati, Basil J Alotaibi, Rami Nassir, Abdulelah Aldhahir, Rakan Ekram, Saeed M Alghamdi, Abdallah Y Naser

**Affiliations:** 1 Pharmacology and Toxicology, Faculty of Medicine, Umm Al-Qura University, Mecca, SAU; 2 Toxicology Center, Ministry of Health, Medina, SAU; 3 Medicine, Ministry of Health, Jeddah, SAU; 4 Family Medicine, Faculty of Medicine, King Abdulaziz University, Jeddah, SAU; 5 Research, Independent Researcher, Riyadh, SAU; 6 Pathology, Umm Al-Qura University, Mecca, SAU; 7 Respiratory Therapy, Faculty of Applied Medical Sciences, Jazan, SAU; 8 School of Public Health and Health Informatics, Umm Al-Qura University, Mecca, SAU; 9 Clinical Technology and Respiratory Care, Faculty of Applied Medical Sciences, Umm Al-Qura University, Mecca, SAU; 10 Applied Pharmaceutical Sciences and Clinical Pharmacy, Faculty of Pharmacoepidemiology, Isra University, Amman, JOR

**Keywords:** toxicology, overdose, drug, substance, toxic

## Abstract

This study aimed to conduct a methodological review of drug-related toxicological studies in Saudi Arabia. A systematic review and a methodological analysis were conducted according to the Preferred Reporting Items for Systematic Reviews and Meta-Analyses (PRISMA) guidelines. Medline and Embase were searched for all types of studies reporting toxicological studies in the English language published until January 10, 2022. The search was conducted using both keywords and Medical Subject Headings (MeSH) terms. The methodological analysis of included studies was assessed using the Newcastle-Ottawa Scale. A total of 3,750 studies were extracted and screened. Of these, 30 observational studies (seven cohort studies and 23 cross-sectional studies) met the inclusion criteria. The methodological scores ranged from five to seven out of 10 possible points. Twelve studies had high quality, and 18 studies had moderate quality. Eight studies focused on adverse drug reactions, eight explored poisoning, four explored drug-related hospitalizations, nine explored drug-induced toxicity, and one explored drug overdose. This research project revealed that most of the drug-related toxicological studies conducted in Saudi Arabia were observational studies of moderate quality. Future studies should focus on the quality of the design and reporting.

## Introduction and background

Clinical toxicology is a branch of toxicology that focuses on the treatment of poisoned patients and the prevention of any toxication due to missed use of any substances. In clinical practice, this field plays a crucial role in toxicological diagnosis, assessment of severity and long-term prognosis, and reversal therapy selection besides managing acute cases [1]. Toxic agents include substances with an environmental component (metals), drugs, industrial by-products (gases, hydrocarbons, and radiation), and essential elements of urban, suburban, or agricultural technology [2].

Medication use has been escalated recently worldwide, and more than 10,000 medications have been approved by the United States Food and Drug Administration (FDA) and, locally, the Saudi National Formulary currently lists over 6,000 pharmaceuticals [3].

Pharmacological agents are chemicals or substances used to treat, halt, or prevent disease, relieve symptoms, and assist in diagnosing ailments. Nevertheless, these medicines are prone to be misused, eventually, negatively influencing patients and causing drug therapy consequences [4]. The most reported consequences associated with drug use are adverse drug reactions, drug interactions, untreated indications, incorrect drug selection, sub-therapeutic dosage, supra-therapeutic dosage, non-compliance, and drug usage without indication [5]. Drug abuse is one of the most severe problems associated with drug use, and recently has become a global concern, with a huge burden on the healthcare system and economical aspects.

Acute poisoning results from deliberate, accidental, or homicidal ingestion of harmful chemicals or drugs into the body, and in some circumstances, this can lead to serious medical problems, including death [6]. Addressing the overall pattern of poisoning would aid in identifying the risk factors and allow for early detection and management of such cases, which should reduce morbidity and mortality. In addition, a major task of toxicological studies is to characterize and explore the toxic level and safety profiles of new drugs, monitor them, and report the mechanism of action of any serious events [7].

Over the past three decades, the fatality rate from drug poisoning has climbed by about 300%, making it the top cause of injury death and a persistent public health problem in the United States; whether illicit or licit, drugs are responsible for around 90% of poisoning deaths [8]. In 2019, 70,630 deaths from the toxic effects of drug poisoning (drug overdose) occurred in the United States [9]. Moreover, the yearly cost of drug-related morbidity and mortality due to non-optimized medication therapy was estimated to be $528.4 billion in 2016, accounting for 16% of United States healthcare spending [10].

In the last years, there has been a large increase in the publication rate of studies in the toxicology field, including studies on reporting adverse drug reactions, medication surveillance, and safety monitoring. However, information on the characteristics and methodological rigor of these studies is unclear. Therefore, in this review, we aimed to explore and evaluate drug-related toxicological studies in Saudi Arabia from a methodological perspective and to identify gaps in the knowledge.

## Review

Methods

Protocol and Registration

This review was conducted in accordance with the Preferred Reporting Items for Systematic Reviews and Meta-Analyses (PRISMA) guidelines to ensure clear and comprehensive reporting [11]. In addition, this study followed the Meta-analysis of Observational Studies in Epidemiology (MOOSE) guidelines [12]. The study protocol was registered in the International Prospective Register of Systematic Reviews (PROSPERO).

Types of Studies Included

All types of original study designs were included in this review.

Search Strategy

We used a comprehensive search strategy developed by an expert researcher to search electronic bibliographic databases. Medline and Excerpta Medica (Embase) databases were searched up to January 10, 2022. A combination of Medical Subject Headings (MeSH) and keywords with both English and American spellings has been used to identify relevant studies. The following search query was used: (toxic* OR drug abuse OR substance abuse OR snake bite OR poison control center OR cannabinoids OR opiate OR amphetamines OR alcohol overdose OR scorpion bite OR drug reaction OR antidote OR overdose OR organophos* OR methamphetamines OR corrosive OR pesticides OR poison*) AND (Saudi Arabia). The entire search strategy for Medline and Embase can be found in Appendices 1 and 2, respectively. Articles were downloaded into EndNote software (V.20, Clarivate, London, UK), and duplicates were removed.

Study Selection

We included original studies that reported outcomes related to drug toxicology in Saudi Arabia. We did not have any restrictions on gender or age to make our conclusion more generalizable and robust. Studies not related to the topic and deemed irrelevant were excluded. Studies that reported non-drug toxicological outcomes were excluded. Randomized trials, case-control studies, cross-sectional studies, and cohort studies were eligible for inclusion in this review, while reviews, case reports, case series, ecologic, in vitro or in vivo studies, guidelines publications, and studies written in languages other than English were excluded.

Data Extraction

The data extraction template included the author's name, title, main aim, the location where the study was carried out, sample size, study design, age, and male-to-female ratio. Data extraction was conducted by one author.

Types of Outcome Measures

The methodological characteristics included the study design, study outcomes, statistical analysis, and sample size of drug-related toxicological studies in Saudi Arabia.

Assessment of Study Quality

The Newcastle-Ottawa Scale was used to evaluate the methodological quality of sampling, selection, exposure, and outcomes of the chosen studies [13]. Using the Newcastle-Ottawa Scale (Appendix 3), each study was assessed on seven criteria divided into three categories: selection of study groups (maximum of five stars), comparability of groups (maximum of two stars), and outcome ascertainment (maximum of three stars). This scale awards a maximum of 10 stars, representing the highest methodological quality. Studies with seven to 10 stars are rated as having high quality, studies with five to six stars are rated as having moderate quality, and studies with less than five stars are rated as having low quality. The Newcastle-Ottawa Scale is still the most commonly used assessment tool in practice as reported by many publications.

Results

Study Selection

**Figure 1:**
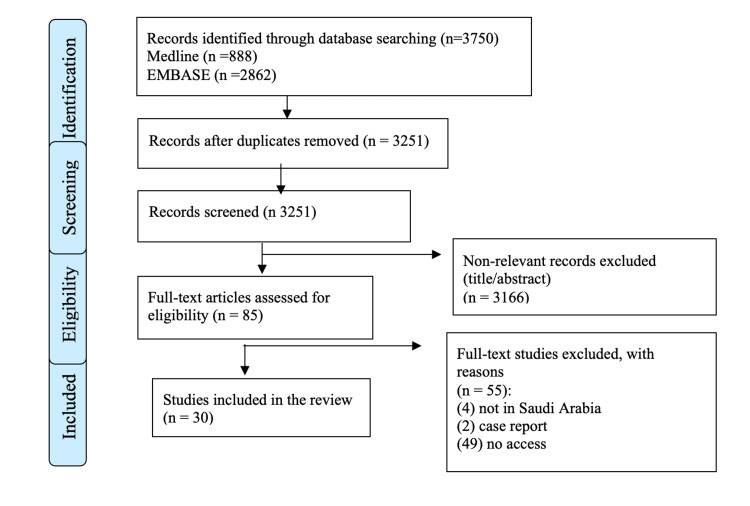
PRISMA flowchart PRISMA: Preferred Reporting Items for Systematic Reviews and Meta-Analyses.

A total of 3,750 studies were identified from our initial databases search (Medline and Embase). After removing duplicates (499), a total of 3,251 studies were screened based on title and abstract. Of these, 3,166 studies were not related to the scope of the review and were excluded, leaving 85 articles to be screened based on the full text. Of these, four studies were from other countries, two were case reports, and we were unable to access 49 studies, which were all excluded. Finally, a total of 30 articles were included in the present systematic review. The article selection process is shown in the flowchart (Figure 1).

General Characteristics of Included Studies

All articles were written in English and conducted in Saudi Arabia. Thirteen studies were from the Riyadh region [3,14-25], nine were from the Makkah region [26-34], three studies were from the Eastern region [35-37], three studies were from the Qassim region [38-40], one study was from the Najran region [41], and one study covered entire Saudi Arabia [42]. In relation to the range of years of publication, the oldest article was from 2012, and the latest was from 2021. The sample size in the included articles ranged between eight and 5,574. The total samples were 25,720. All subjects included in the study were related to toxicology. Studies can be divided into four groups based on the age of the samples. The first group was comprised only of adolescents, the second group was comprised only of children, the third group was comprised only of neonates, and the last group included samples of different ages. Most studies included both men and women, except two studies that included only women. Out of 30 studies, four studies were aimed to explore drug-related hospitalizations [3,32,35,38], eight studies explored the adverse drug reactions [14,15,17,21,33,37,40,42], three studies explored drug-induced cardiotoxicity [16,24,27], four studies explored drug-induced nephrotoxicity [19,23,30,31], one study explored drug-induced pulmonary toxicity [26], one study explored drug-induced retinal toxicity [20], eight studies explored poisoning [18,22,25,28,29,34,39,41], and one study explored drug overdose [36]. Full details about the included studies are available in Table 1.

**Table 1:** Characteristics of included studies ADRs: adverse drug reactions; DRPs: drug-related problems; HCQ: hydroxychloroquine; VAN: vancomycin; TZP: tazobactam; MEM: meropenem; ADEs: adverse drug events; HER2: human epidermal growth factor receptor-2; TNF: tumor necrosis factor; TEN: toxic epidermal necrolysis; SJS: Stevens-Johnson syndrome.

Author	Title	Main aims	Region	Study design	Sample size	Age	Sex (M/F)
Alghamdy et al. [35]	Admissions for drug-related problems at the emergency department of a university hospital in the Kingdom of Saudi Arabia	Estimate the prevalence of admissions as a result of DRPs at the emergency department (ED)	Eastern Saudi Arabia	Cross-sectional study	5,574	NA	NA
Alayed et al. [38]	Adverse drug reaction (ADR) as a cause of hospitalization at a government hospital in Saudi Arabia: a prospective observational study	Evaluate ADR-related admissions at King Saud Hospital	Qassim	Cross-sectional study	4,739	±49.08 years	52.60%
Al-Malaq et al. [14]	Adverse drug reactions caused by methotrexate in Saudi population	Document adverse drug reactions (ADRs) of methotrexate (MTX) in Saudi patients	Riyadh	Cross-sectional study	186	Group 1 (45.89 years), Group 2 (51.51 years)	14.50%
Abu Esba et al. [15]	Adverse drug reactions spontaneously reported at a tertiary care hospital and preventable measures implemented	Characterize and describe ADR reports at one of the largest healthcare institutions in the region and share the measures implemented by the team	Riyadh	Cross-sectional study	1,156	≤18 years (27.5%), 19-64 years (54.3%), >65 years (18.2%)	42.70%
Al-Jizani et al. [26]	Bleomycin pulmonary toxicity in adult Saudi patients with Hodgkin’s lymphoma	Describe bleomycin pulmonary toxicity (BPT) in Hodgkin’s lymphoma (HL) patients treated with bleomycin-containing chemotherapy regimens	Makkah	Cross-sectional study	164	24 years	54%
Alkofide et al. [16]	Cardiotoxicity and cardiac monitoring among anthracycline-treated cancer patients: a retrospective cohort study	Estimate the incidence and determinants of anthracycline-induced cardiotoxicity, both acute and chronic	Riyadh	Cohort study	235	<60 years (74.5%), >60 years (25.5%)	54.90%
Hamed et al. [27]	Cardiotoxicity of the adjuvant trastuzumab in a Saudi population: clinical experience of a single institution	Evaluate the cardiac safety of trastuzumab in clinical practice	Makkah	Retrospective observational study	57	<40 years (25.2%), >40 years (74.8%)	0%
Abanmy et al. [17]	Clozapine-induced blood dyscrasias in Saudi Arab patients	Report the incidence of clozapine-induced hematologic toxicity in Saudi Arabian patients	Riyadh	Cross-sectional study	147	38 ± 11.42 year	52%
Alzahrani et al. [28]	Drug poisoning and associated factors in western Saudi Arabia: a five-year retrospective chart review (2011-2016)	Explore drug-poisoning prevalence patterns, associated risk factors (gender, age, and exposure circumstances), and outcomes in western Saudi Arabia	Makkah	Cross-sectional study	1,474	0-4 years (51.8%), 5-14 years (8.8%), 15-24 years (17.5%), >24 years (21.9%)	40%
Al-Arifi et al. [3]	Emergency department visits and admissions due to drug related problems at Riyadh Military Hospital (RMH), Saudi Arabia	Determine the incidence and types of emergency department visits and admissions due to drug-related problems at Riyadh Military Hospital, to assess the severity and preventability of these drug-related admissions or visits, and identify the drugs and patient groups that are most commonly involved	Riyadh	Prospective cohort observational study	300	51	53.33%
Alruwaili et al. [18]	An epidemiological snapshot of toxicological exposure in children 12 years of age and younger in Riyadh	Describe the characteristics of acute poison exposure and related therapeutic interventions in children aged 12 years and younger	Riyadh	Prospective, descriptive, cross-sectional study	1,035	27 months	Males = 51%; females = 38%, unidentified = 18%
Tobaiqy et al. [29]	Frequency and management of acute poisoning among children attending an emergency department in Saudi Arabia	Evaluate the frequency and management of acute poisoning among children attending the emergency room at East Jeddah Hospital, Jeddah city, Saudi Arabia	Makkah	Retrospective medical chart review	69	0-5 years (59.4%), 6-11 years (26.1%), 12-16 years (14.5%)	55.10%
Omrani et al. [19]	High dose intravenous colistin methanesulfonate therapy is associated with high rates of nephrotoxicity; a prospective cohort study from Saudi Arabia	Evaluate colistin-associated nephrotoxicity rates in our institution and explore risk factors in our patient population	Riyadh	Prospective cohort study	67	57.48 years	67.20%
Al Adel et al. [20]	Hydroxychloroquine dosing and toxicity: a real-world experience in Saudi Arabia of 63 patients	Report the percentage of patients on high daily doses per weight (>5 mg/kg) of HCQ, as per the latest American Academy of Ophthalmology (AAO) screening guidelines for HCQ toxicity, and look at the percentage of retinal toxicity	Riyadh	Cross-sectional study	63	45 ± 13.5	8%
Almutairy et al. [30]	Impact of colistin dosing on the incidence of nephrotoxicity in a tertiary care hospital in Saudi Arabia	Incidence of colistin-associated acute kidney injury (AKI)	Makkah	Retrospective chart review study	198	55.7 ± 19.36 years	62%
Tookhi et al. [31]	Impact of combining vancomycin with piperacillin/tazobactam or with meropenem on vancomycin-induced nephrotoxicity	Evaluate acute kidney injury rates in patients receiving VAN with either TZP or MEM	Makkah	Retrospective cohort study	158	Group 1 (56 years), Group 2 (53 years)	46.80%
Wahba et al. [41]	Incidence and profile of acute intoxication among adult population in Najran, Saudi Arabia: a retrospective study	Determine the extent of acute adult intoxication among the population located in the Najran area, Saudi Arabia, over the last 3 years (from January 2017 to December 2019)	Najran	Retrospective observational study	852	15-25 years (53.87%), 26-35 years (25.47%), >35 years (20.66%)	46.50%
Almalag et al. [21]	Incidence of hemorrhagic cystitis after cyclophosphamide therapy with or without mesna: a cohort study and comprehensive literature review	Investigate the incidence of hemorrhagic cystitis in patients receiving cyclophosphamide therapy with or without mesna	Riyadh	Retrospective chart review	718	Group 1 (49 years), Group 2 (50 years)	27.40%
Aldardeer et al. [32]	Medications related emergency admissions: causes and recommendations	Investigate the incidence of ADEs leading to hospitalization and evaluate the severity and factors contributing to medication-related emergency admissions at King Faisal Specialist Hospital and Research Center, Jeddah, Saudi Arabia	Makkah	Retrospective study	698	55	53.30%
Almansori et al. [36]	Paracetamol overdose: analysis of a sample from a tertiary hospital in Eastern Saudi Arabia	Characterize patients in a sample from the Kingdom of Saudi Arabia (KSA) with a paracetamol overdose	Eastern Saudi Arabia	Retrospective chart review	86	24.4 ± 6.6	28%
Tobaiqy et al. [33]	Parental experience of potential adverse drug reactions related to their oral administration of antipyretic analgesic medicines in children in Saudi Arabia	Explore parental experiences of potential ADRs related to their oral administration of antipyretic analgesics in children in the Kingdom of Saudi Arabia	Makkah	Cross-sectional survey	661	<5 years (29%), 5-10 years (58.9%), ≥10 years (12.1%)	M = 27.8%, F = 63.8%, not disclosed = 8.3%
Bakhaidar et al. [34]	Pattern of drug overdose and chemical poisoning among patients attending an emergency department, western Saudi Arabia	Describe the current pattern and assess risk factors of drug overdose and chemical poisoning in King Khalid National Guard Hospital, Jeddah	Makkah	Retrospective chart review study	129	<12 years (44.2%), 12-35 years (33.3%) >35 years (22.5%)	45.70%
Alnasser et al. [39]	Pattern of food, drug and chemical poisoning in Qassim region, Saudi Arabia from January 2017 to December 2017	Determine and analyze the pattern of poisoning cases induced by food, drugs, and chemicals reported to the Department of Environmental and Occupational Health in Qassim province in the Kingdom of Saudi Arabia	Qassim	Retrospective cross-sectional study	381		44%
Alghadeer et al. [22]	The patterns of children poisoning cases in community teaching hospital in Riyadh, Saudi Arabia	Identify the most common classes of toxic substances and route of poisoning in children and investigate the pattern of drug and chemical poisoning in suspected case fatalities, the subsequent need for hospital admission, and arrival time to hospital	Riyadh	Retrospective cross-sectional	735	2.7 years	Male = 45.8%, female = 49.7%, undocumented gender = 4.5%
Abouelkheir et al. [23]	Pediatric acute kidney injury induced by concomitant vancomycin and piperacillin-tazobactam	Report cases of drug-induced nephrotoxicity that have occurred in children who were admitted to the hospital and received concomitant therapy with vancomycin and piperacillin-tazobactam	Riyadh	Retrospective cohort evaluative	8	5.1 years	50%
Almubark et al. [42]	Population-based estimates of community-based adverse drug reactions (ADRs) in the Kingdom of Saudi Arabia	Estimate population-based rates of ADRs in the community in the Kingdom of Saudi Arabia (KSA)	Saudi Arabia	Cross-sectional study	5,228	18-36 years (50.23%), 37-56 years (37.51%), 57+ years (12.26%)	50.17%
Abdel-Razaq et al. [24]	Risk factors associated with trastuzumab-induced cardiotoxicity in patients with human epidermal growth factor receptor 2-positive breast cancer	Explore risk factors associated with the development of cardiotoxicity in patients with HER2-positive breast cancer	Riyadh	Retrospective study	146	52.7	0.00%
AlAskar et al. [37]	Risk of neutropenia in inflammatory bowel disease patients treated with TNF inhibitors: a single-center, retrospective cohort study	Ascertain the relationship between the use of TNF inhibitors and the development of neutropenia in patients with inflammatory bowel disease (IBD)	Eastern Saudi Arabia	Retrospective cohort study	281	Group1 (32.90 years), Group 2 (32.61 years)	53%
Alanazi et al. [25]	Severity scores and their associated factors among orally poisoned toddlers: a cross sectional single poison center study	Evaluate the poison severity score and its associated factors among toddlers with orally ingested substances at a pediatric emergency department (ED) in central Saudi Arabia	Riyadh	Retrospective cross-sectional	165	1-2 years (72.1%), 2-3 years (27.9%)	58%
Alajaji et al. [40]	Toxic epidermal necrolysis (TEN)/Stevens-Johnson syndrome (SJS) epidemiology and mortality rate at King Fahad Specialist Hospital (KFSH) in Qassim region of Saudi Arabia: a retrospective study	Study the epidemiology of SJS/TEN and associated mortality rate in the Qassim region, Saudi Arabia	Qassim	Retrospective cohort study	10	16-36 years (50%), 37-57 years (30%), >57 years (20%)	40%

Methodological Characteristics of Included Studies

All 30 articles included in this systematic review were observational studies (seven cohort studies and 23 cross-sectional studies). Of the 30 studies, 26 (86.66%) obtained their data from an administrative database and/or patient interviewing [3,14-18,21-24,26,28-32,34-41], two studies (6.66%) from a survey [33,42], and two studies (6.66%) did not specify the data source [19,27]. Most of the studies reported the outcomes over a short period. In addition, gender variation in the reporting of the studies was very limited. The details of the study characteristics are listed in Table 1.

Quality of Included Studies

The included studies were assessed for methodological quality using the Newcastle-Ottawa Scale. Among the 30 included studies, 12 studies scored seven stars, which means they had a high quality. Eighteen studies scored five to six stars, which means a moderate quality, and none were of low quality. The detailed scoring is shown in Table 2.

**Table 2:** Quality of included studies

Author								
	Selection				Comparability	Outcome		Total quality score
	Representativeness of the sample	Sample size	Non-respondents	Ascertainment of exposure	The subjects in different outcome groups are comparable, based on the study design or analysis. Confounding factors are controlled	Assessment of the outcome	Statistical test	
Alghamdy et al. [35]	*	*		**	__	**		6
Alayed et al. [38]	*	*		**	__	**	*	7
Al-Malaq et al. [14]	*			**	__	**	*	6
Abu Esba et al. [15]	*	*		**	__	**	*	7
Al-Jizani et al. ​​​​​​​[26]	*	*		**	__	**	*	7
Alkofide et al. ​​​​​​​[16]	*	*		**	__	**	*	7
Hamed et al. ​​​​​​​[27]	*			**	__	**	*	6
Abanmy et al. ​​​​​​​[17]	*			**	__	**		5
Alzahrani et al. ​​​​​​​[28]	*	*		**	__	**	*	7
Al-Arifi et al. ​​​​​​​[3]	*			**		**		5
Alruwaili et al. ​​​​​​​[18]	*	*		**	__	**		6
Tobaiqy et al. ​​​​​​​[29]	*			**	__	**		5
Omrani et al. ​​​​​​​[19]	*			**	__	**		5
Al Adel et al. ​​​​​​​[20]	*			**	__	**		5
Almutairy et al. ​​​​​​​[30]	*	*		**	__	**	*	7
Tookhi et al. ​​​​​​​[31]	*	*		**	__	**	*	7
Wahba et al. ​​​​​​​[41]	*	*		**	__	**	*	7
Almalag et al. ​​​​​​​[21]	*	*		**	__	**	*	7
Aldardeer et al. ​​​​​​​[32]	*	*		*	__	**		5
Almansori et al. ​​​​​​​[36]	*			**	__	**		5
Tobaiqy et al. ​​​​​​​[33]	*	*		**	__	**		6
Bakhaidar et al. ​​​​​​​[34]	*			**	__	**		5
Alnasser et al. ​​​​​​​[39]	*	*		**	__	**		6
Alghadeer et al. ​​​​​​​[22]	*	*		**	__	**		6
Abouelkheir et al. ​​​​​​​[23]	*			**	__	**		5
Almubark et al. ​​​​​​​[42]	*	*		**	__	**	*	7
Abdel-Razaq et al. ​​​​​​​[24]	*	*		**	__	**	*	7
AlAskar et al. ​​​​​​​[37]	*	*		**	__	**	*	7
Alanazi et al. ​​​​​​​[25]	*			**	__	**	*	6
Alajaji et al. ​​​​​​​[40]	*			**	__	**		5

Discussion

To the best of our knowledge, this is the first study to evaluate toxicological studies in Saudi Arabia from a methodological perspective. The findings of this study show that all studies published within the last 10 years were primarily observational in nature (seven cohort studies and 23 cross-sectional studies). According to the Newcastle-Ottawa Scale, 12 studies had high quality, and 18 studies had moderate quality.

Administratively, Saudi Arabia is divided into 13 regions. The majority of the studies were conducted in Riyadh (43.3%) and Makkah (30%) regions. This could be attributed to the fact that those two regions have better and more healthcare facilities, the most prominent universities, and an increased population than other regions in Saudi Arabia, which might facilitate more research to be conducted.

The term "methodological quality" relates to all parts of a study, including design, conduct, analysis, and reporting of results that impact the study's capability to answer the question addressed adequately [43]. Limitations in any part of the study’s design, conduct, analysis, and reporting of results contribute to the "risk of bias" or lower methodological quality. Furthermore, research data that produce negative results are not always reported, resulting in publication bias [43]. However, given the nature of the specialty (toxicology), most of the studies are expected to be reporting events or medication misuse of safety.

Most of the studies included in this review (86.66%) gathered their data from local health institute databases and/or patient interviews. Using administrative databases/registries for observational studies has several advantages, including considerable sample size. These registries can boost the study's validity, reduce standard error, improve precision in identifying any effect, and be highly representative of the general population [44]. When compared to research using alternative data sources, the pre-existing and continual accumulation of patient information in an administrative database/registry, whose primary goal is to record health information, saves time, money, and human resources in the data collection process. On the other hand, administrative databases/registries for identifying cases and exposures have limitations. Significant disparities in diagnosis may compromise the validity of the study results if there is misclassification or changes in disease coding over time. The coding accuracy varies depending on the conditions, databases, and registries [45].

In this review, all the studies included were observational in nature. This could be attributed to the objectives of this study, as drug-related toxicity studies are usually conducted for monitoring the drugs, and in most cases, these studies are usually cross-sectional or post-marketing cohort studies [46]. In addition, another reason that could be attributed to these findings is the low volume and rate of published randomized clinical trials in Saudi Arabia. Furthermore, observational studies have the advantage of being usually more inexpensive, relatively rapid, and more accessible than randomized controlled trials.

The main strength of observational studies over randomized controlled trials is their more excellent proximity to "real-life situations," as randomized controlled trials have stricter inclusion criteria and strict protocols that may not reflect clinical practice, especially when investigating safety or toxicology-related outcomes. However, it is important to highlight that observational studies have more significant variability of medical interventions and patient populations that are more representative of clinical practice. They can be used to investigate rare outcomes and uncover unusual adverse effects [47]. Hence, the previously mentioned advantages might be the reason for conducting observational studies, as shown in our methodological review [48].

Toxicologists should carefully select the titles, abstracts, and keywords of their studies to ensure access and retrieval in literature reviews of the research and provide study protocol in sufficient detail to allow reviewers to assess methodological quality. This, in turn, recommends that future researchers should be trained properly in choosing study design, sample size, appropriate statistical analysis, and proper reporting to ensure high-quality research that would help policymakers to make the right decision. Furthermore, improving the knowledge of the physicians with respect to drug-related problems as well as education of the community regarding the risk of medication misuse could help in the improvement of patient safety. Future studies should also focus on highlighting gender variation in toxicological outcomes.

This study has several notable strengths. It is the first study to evaluate toxicological studies in Saudi Arabia from a methodological perspective, including a comprehensive literature search in two relevant databases (Embase and Medline) and proper screening of studies that meet the eligibility criteria. The review's conformance with established guidelines for systematic literature reviews is an additional strength of this review (i.e., PRISMA statement). In addition, we used the Newcastle-Ottawa Scale, which is a widely used and endorsed tool by the Cochrane Collaboration. The study protocol was registered with PROSPERO to provide openness and allow replication or modifications in the future.

Most of the information in toxicology-related databases is not available to the general public. Other considerations include confidential data that would not be exchangeable. Furthermore, the lack of MeSH terms to categorize toxicology makes retrieving toxicological data from search engines challenging [43]. This limited and difficult access could be a reason behind the lack of national data.

However, there are certain limitations to this study. First, this methodological review has a limitation in that we only searched publications in two relevant databases (Embase and Medline), which means we may not have included all prospective toxicological studies. However, Embase and Medline are considered the largest medical databases and they include most of the studies in the literature; therefore, we assume that we have not missed important studies in the literature. Even though this review was intended to cover a broad spectrum of literature, it is limited to current publications from the last 10 years. This was attributed to time constrain.

## Conclusions

The present paper systematically evaluated the methodological quality of the studies related to toxicology published in Saudi Arabia during the last 10 years. All the studies included in this review were cross-sectional and cohort studies. Based on the score stratification approach we used to categorize research based on their final ratings, most of the studies in our review demonstrated moderate methodological strength.

## Appendices

Appendix 1

**Table 3:** Medline search strategy (until January 10, 2022)

1.	Toxic.mp. or exp Plants, Toxic/
2.	Toxic*.mp. [mp=title, abstract, original title, name of substance word, subject heading word, floating sub-heading word, keyword heading word, organism supplementary concept word, protocol supplementary concept word, rare disease supplementary concept word, unique identifier, synonyms]
3.	drug abuse.mp. or exp substance-related disorders/
4.	substance abuse.mp. [mp=title, abstract, original title, name of substance word, subject heading word, floating sub-heading word, keyword heading word, organism supplementary concept word, protocol supplementary concept word, rare disease supplementary concept word, unique identifier, synonyms]
5.	snake bite.mp. or exp Snake Bites/
6.	poison control center.mp. or exp Poison Control Centers/
7.	cannabinoids.mp. or exp Cannabinoids/
8.	opiate.mp. or exp Opiate Alkaloids/
9.	amphetamines.mp. or exp Amphetamines/
10.	exp Poisoning/ or exp Drug Overdose/ or alcohol overdose.mp.
11.	exp Scorpion Venoms/ or exp Scorpions/ or exp Scorpion Stings/ or scorpion bite.mp.
12.	exp "Drug-Related Side Effects and Adverse Reactions"/ or exp Drug Hypersensitivity/ or drug reaction.mp.
13.	antidote.mp. or exp Antidotes/
14.	exp Opiate Overdose/ or exp Drug Overdose/ or overdose.mp.
15.	organophos.mp. or exp Organophosphorus Compounds/
16.	organophos*.mp. [mp=title, abstract, original title, name of substance word, subject heading word, floating sub-heading word, keyword heading word, organism supplementary concept word, protocol supplementary concept word, rare disease supplementary concept word, unique identifier, synonyms]
17.	methamphetamines.mp. or exp Methamphetamine/
18.	corrosive.mp. or exp Caustics/
19.	pesticides.mp. or exp Pesticides/
20.	poison.mp. or exp Poisons/
21.	poison*.mp. [mp=title, abstract, original title, name of substance word, subject heading word, floating sub-heading word, keyword heading word, organism supplementary concept word, protocol supplementary concept word, rare disease supplementary concept word, unique identifier, synonyms]
22.	1 or 2 or 3 or 4 or 5 or 6 or 7 or 8 or 9 or 10 or 11 or 12 or 13 or 14 or 15 or 16 or 17 or 18 or 19 or 20 or 21
23.	Saudi Arabia.mp. [mp=title, abstract, original title, name of substance word, subject heading word, floating sub-heading word, keyword heading word, organism supplementary concept word, protocol supplementary concept word, rare disease supplementary concept word, unique identifier, synonyms]
24.	22 and 23

Appendix 2

**Table 4:** Embase search strategy (until January 10, 2022)

1.	exp toxic dose/ or toxic.mp. or exp toxic octopus/ or exp toxic substance/
2.	toxic*.mp. [mp=title, abstract, heading word, drug trade name, original title, device manufacturer, drug manufacturer, device trade name, keyword heading word, floating subheading word, candidate term word]
3.	drug abuse.mp. or exp drug abuse/
4.	substance abuse.mp. or exp substance abuse/
5.	snake bite.mp. or exp snakebite/
6.	poison control center.mp. or exp poison center/
7.	cannabinoids.mp. or exp cannabinoid/
8.	exp opiate addiction/ or opiate.mp. or exp opiate/ or exp opiate overdose/
9.	amphetamines.mp. or exp amphetamine derivative/
10.	exp alcohol intoxication/ or exp intoxication/ or exp drug overdose/ or exp alcohol/ or alcohol overdose.mp.
11.	scorpion bite.mp. or exp scorpion sting/
12.	exp adverse drug reaction/ or exp drug hypersensitivity/ or drug reaction.mp. or exp drug eruption/
13.	exp antidote/ or antidote.mp.
14.	overdose.mp. or exp intoxication/
15.	exp toxicology/ or exp pesticide/ or organophos.mp. or exp organophosphate/
16.	organophos*.mp. [mp=title, abstract, heading word, drug trade name, original title, device manufacturer, drug manufacturer, device trade name, keyword heading word, floating subheading word, candidate term word]
17.	exp methamphetamine dependence/ or exp methamphetamine/ or methamphetamines.mp.
18.	corrosive.mp. or exp chemical burn/ or exp corrosion/
19.	pesticides.mp. or exp pesticide/
20.	exp poison center/ or exp poison/ or poison.mp.
21.	poison*.mp. [mp=title, abstract, heading word, drug trade name, original title, device manufacturer, drug manufacturer, device trade name, keyword heading word, floating subheading word, candidate term word]
22.	1 or 2 or 3 or 4 or 5 or 6 or 7 or 8 or 9 or 10 or 11 or 12 or 13 or 14 or 15 or 16 or 17 or 18 or 19 or 20 or 21
23.	Saudi Arabia.mp. or exp Saudi Arabia/
24.	22 and 23

Appendix 3

Newcastle-Ottawa Scale Adapted for Cross-Sectional Studies

Selection: (Maximum five stars)

(1) Representativeness of the sample:

(a) Truly representative of the average in the target population. * (All subjects or random sampling)

(b) Somewhat representative of the average in the target population. * (Non-random sampling)

(c) Selected group of users.

(d) No description of the sampling strategy.

(2) Sample size:

(a) Justified and satisfactory. *

(b) Not justified.

(3) Non-respondents:

(a) Comparability between respondent and non-respondent characteristics is established, and the response rate is satisfactory. *

(b) The response rate is unsatisfactory, or the comparability between respondents and non-respondents is unsatisfactory.

(c) No description of the response rate or the characteristics of the responders and the non-responders.

(4) Ascertainment of the exposure (risk factor):

(a) Validated measurement tool. **

(b) Non-validated measurement tool, but the tool is available or described. *

(c) No description of the measurement tool.

Comparability: (Maximum two stars)

(1) The subjects in different outcome groups are comparable, based on the study design or analysis. Confounding factors are controlled.

(a) The study controls for the most important factor (select one). *

(b) The study control for any additional factor. *

Outcome: (Maximum three stars)

(1) Assessment of the outcome:

(a) Independent blind assessment. **

(b) Record linkage. **

(c) Self-report. *

(d) No description.

(2) Statistical test:

(a) The statistical test used to analyze the data is clearly described and appropriate, and the measurement of the association is presented, including confidence intervals and the probability level (p-value). *

(b) The statistical test is not appropriate, not described, or incomplete.
